# Supplementary figures and images for: Host influence on the eukaryotic virome of sympatric mosquitoes and abundance of diverse viruses with a broad host range
Source: PLoS One. 2024 Apr 30;19(4):e0300915. doi: 10.1371/journal.pone.0300915 (PMC11060559; doi:10.1371/journal.pone.0300915)

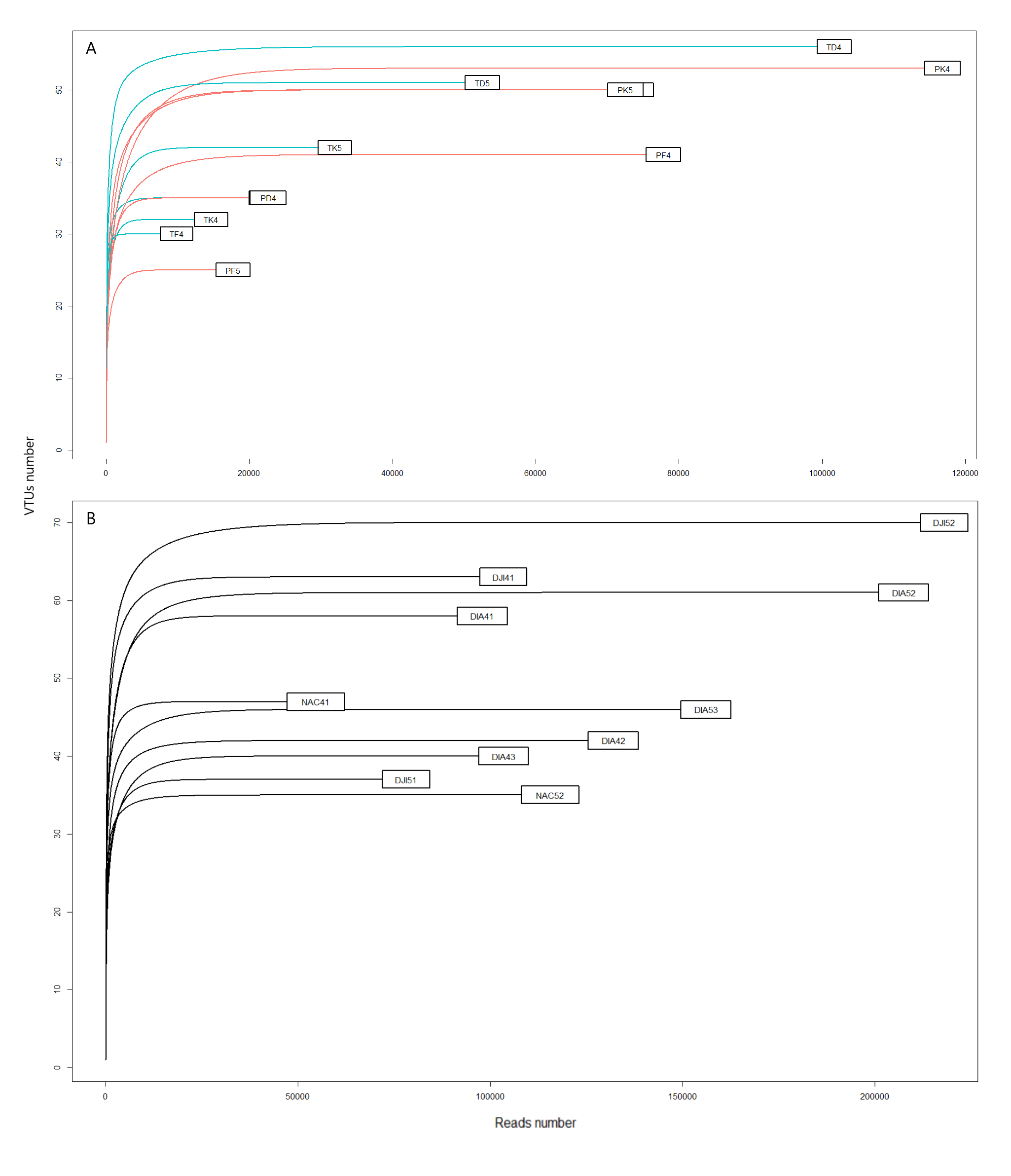

Supplement: S1 Fig — The x-axis shows the number of virus-like reads, and the y-axis the number of viral taxonomic units (VTUs) per library. (A) Libraries of Culex poicilipes (red) and Culex tritaeniorhynchus (blue). (B) Libraries of Aedes vexans. (TIF) [file pone.0300915.s002.tif]

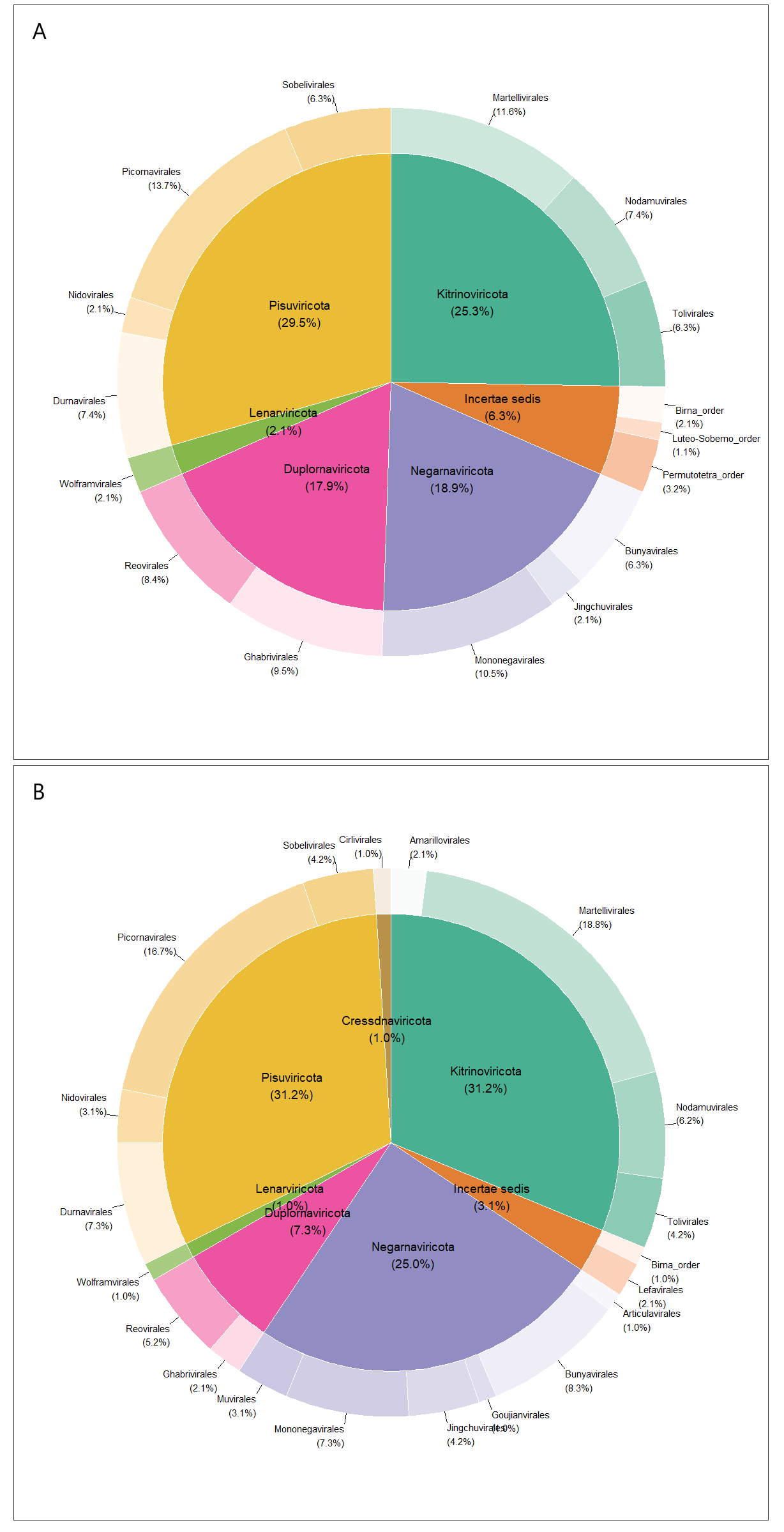

Supplement: S2 Fig — Distribution of viral taxonomic units (VTUs) among orders (external donut chart) and phyla (inner pie chart) found in (A) Culex poicilipes and Culex tritaeniorhynchus, and (B) Aedes vexans. Percentages between brackets represent the proportion of all VTUs in each order or phyla. The term “Incertae sedis” stands for taxa whose classification is still undefined at the phylum level. (TIF) [file pone.0300915.s003.tif]

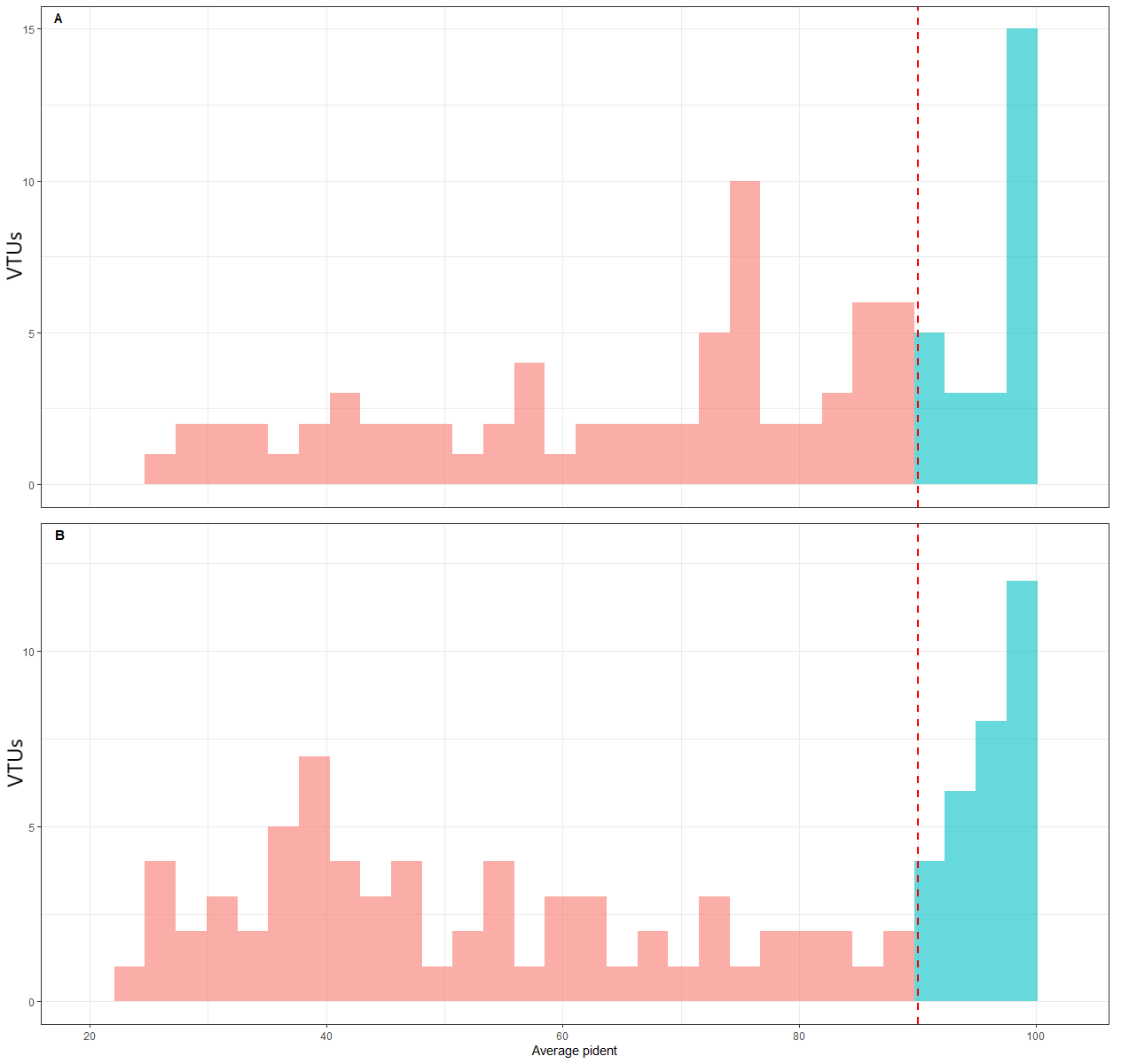

Supplement: S3 Fig — Average percent identities at the amino acid level from all the contigs of each viral taxonomic units (VTUs) with their best hit in the viromes of (A) Culex mosquitoes and (B) Aedes vexans. The bars in blue indicate an average percent identity higher than 90% and thus VTUs likely including sequences of the virus species found as their best hit. The red bars represent VTUs with less than 90% identity to their best hit and thus probably involving sequences of new virus species. (TIF) [file pone.0300915.s004.tif]

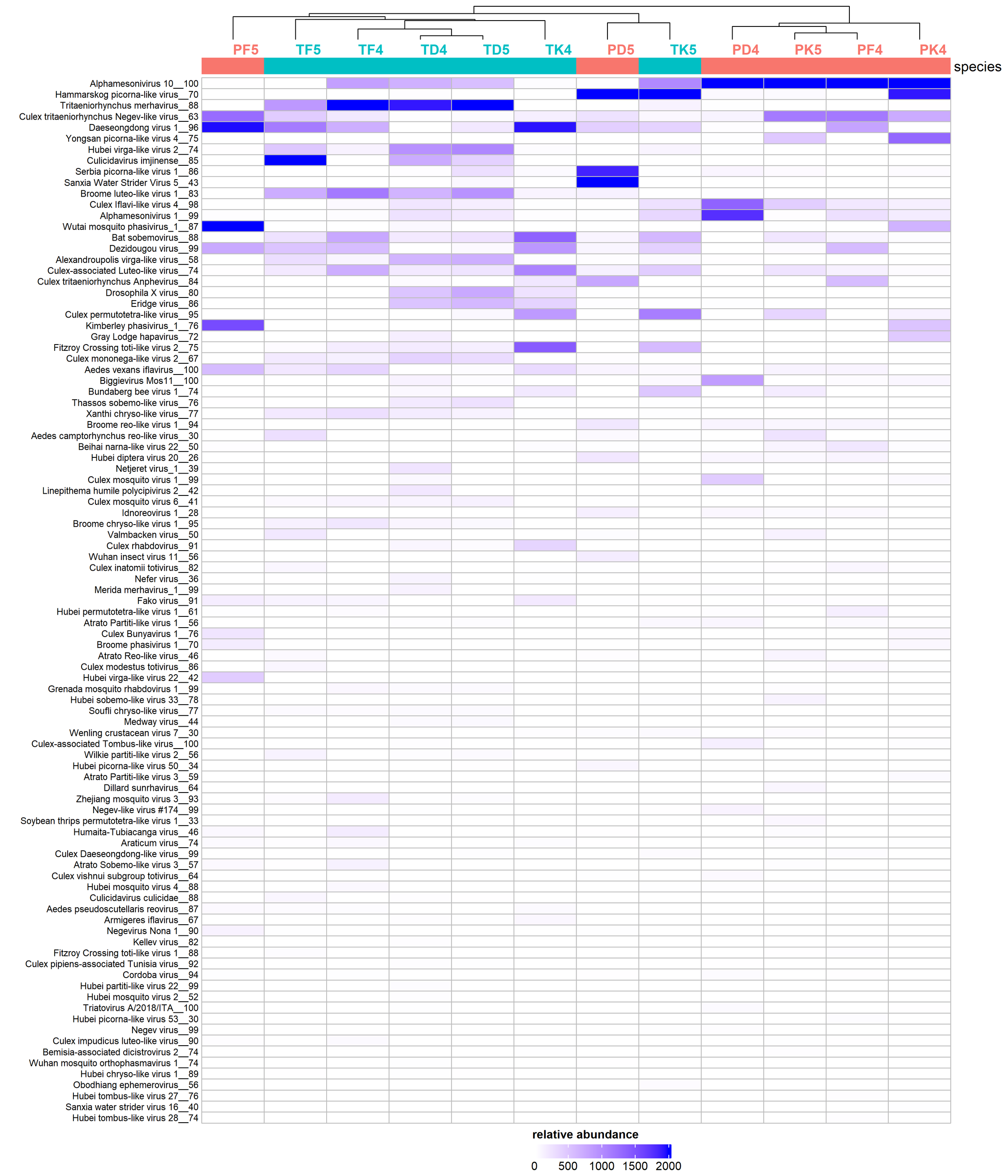

Supplement: S4 Fig — Library names are indicated on top of the heatmap (see Table 1 for explanation of acronyms), along with a hierarchical clustering, and library colour indicates mosquito species (red: Culex poicilipes, blue: Culex tritaeniorhynchus). Tile colour stands for read abundance; the more abundant a cluster, the warmer the colour. The VTUs are ranked following total read abundance. (TIF) [file pone.0300915.s005.tif]

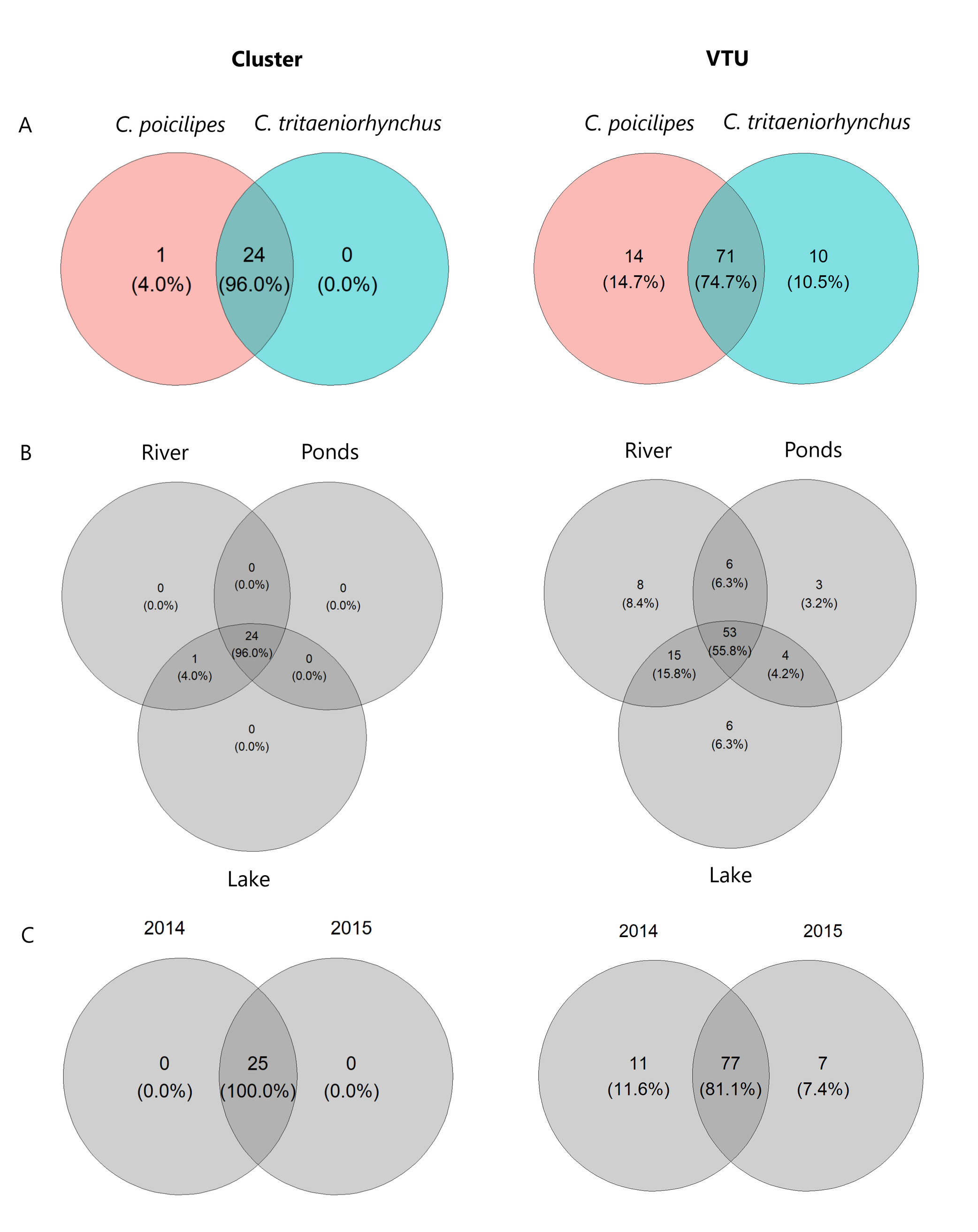

Supplement: S5 Fig — Distribution of clusters (left) and viral taxonomic units (right) between mosquito species (A), sites (B) and years (C). Numbers between brackets stand for the proportion of each group among the total number of taxa. (TIF) [file pone.0300915.s006.tif]

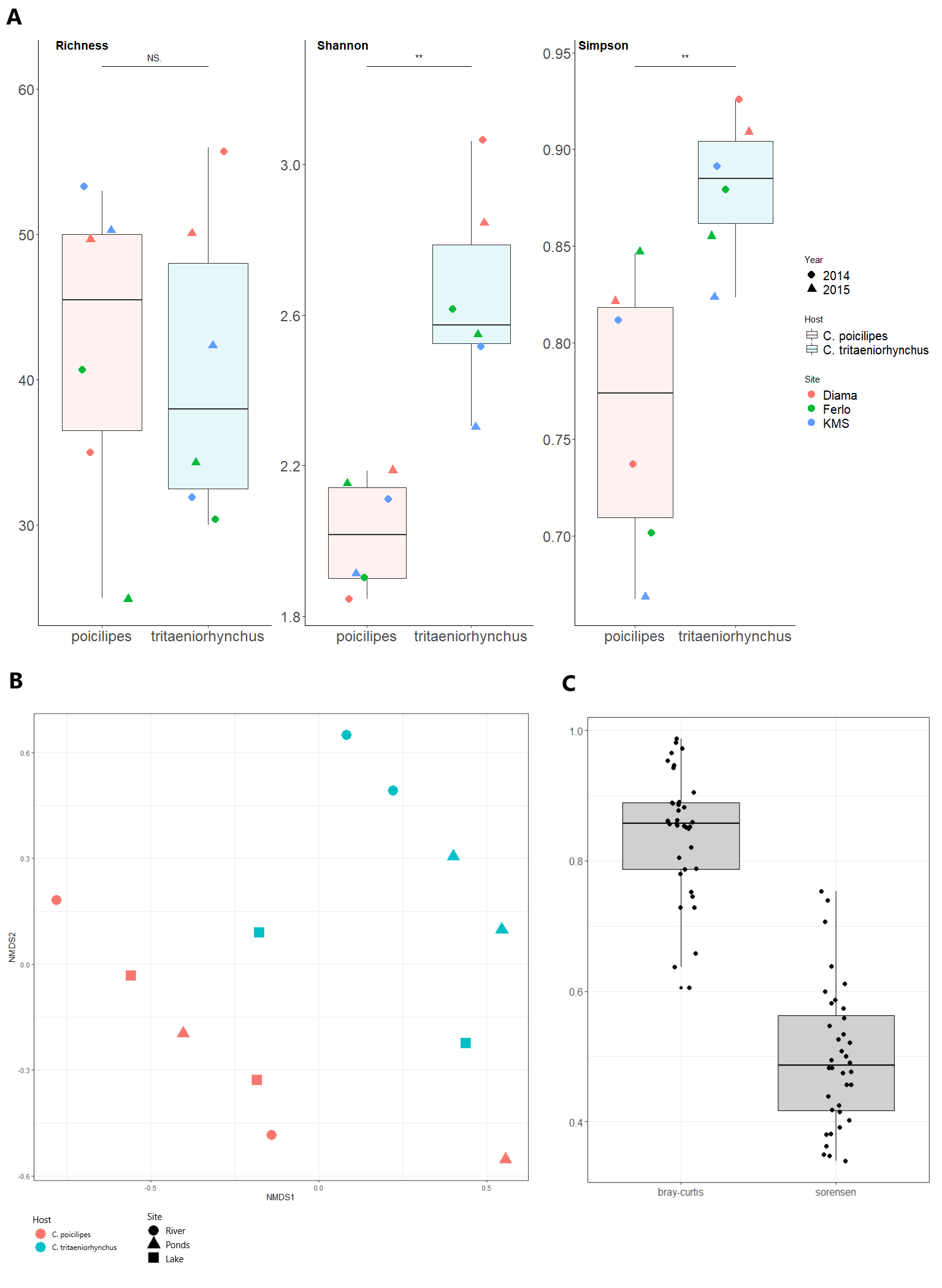

Supplement: S6 Fig — A. Distribution of cluster richness, Shannon and Simpson indices between libraries of Culex poicilipes (in red) and libraries of Culex tritaeniorhynchus (in blue). Dot color indicates the habitat while dot shape represents year. The significance of the comparison between distributions of the two species is shown above boxplots (Wilcoxon Mann-Whitney test). B. Non-metric multidimensional scale with Bray-Curtis dissimilarities obtained from the viromes of the two Culex species. Dot color indicates mosquito species and dot shape represents habitat. C. Comparison of Sorensen (for presence-absence data) and Bray-Curtis (for abundance data) dissimilarities between libraries. Each point therefore represents a dissimilarity index value between two libraries belonging each to a different mosquito species. (TIF) [file pone.0300915.s007.tif]

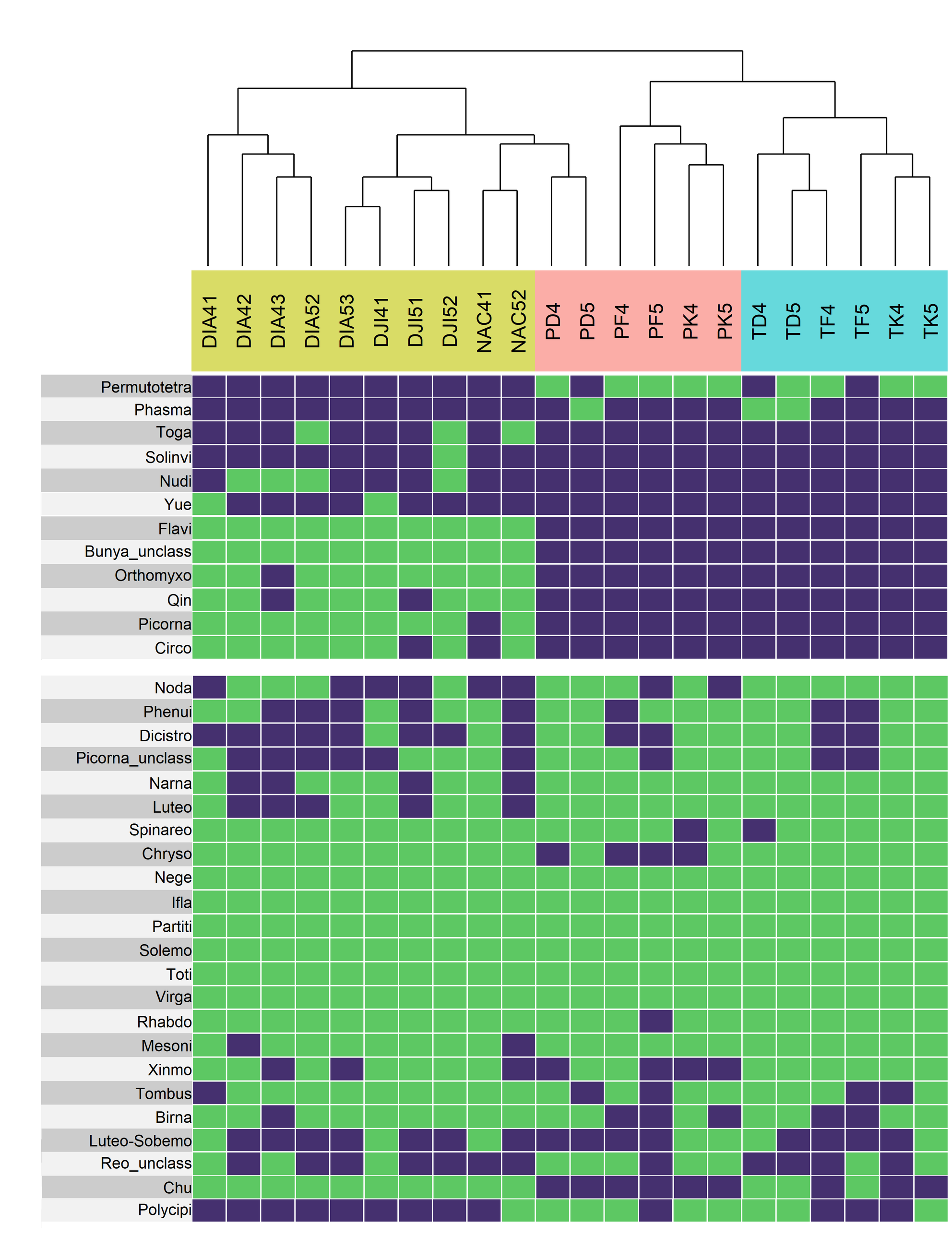

Supplement: S7 Fig — Heatmap showing the presence (green) or absence (blue) of clusters in the different libraries. Libraries are ranked on the x axis following a hierarchical clustering (dendrogram available on top of the heatmap). Library names are coloured following mosquito species, with libraries from Aedes vexans shown in yellow, Culex poicilipes in red and Culex tritaeniorhynchus in blue (see Table 1 for explanation of acronyms). To facilitate visualization of shared clusters, the heatmap is separated into a top panel with the clusters only present in either the Aedes or the Culex species, and a bottom panel with the shared clusters. (TIF) [file pone.0300915.s008.tif]

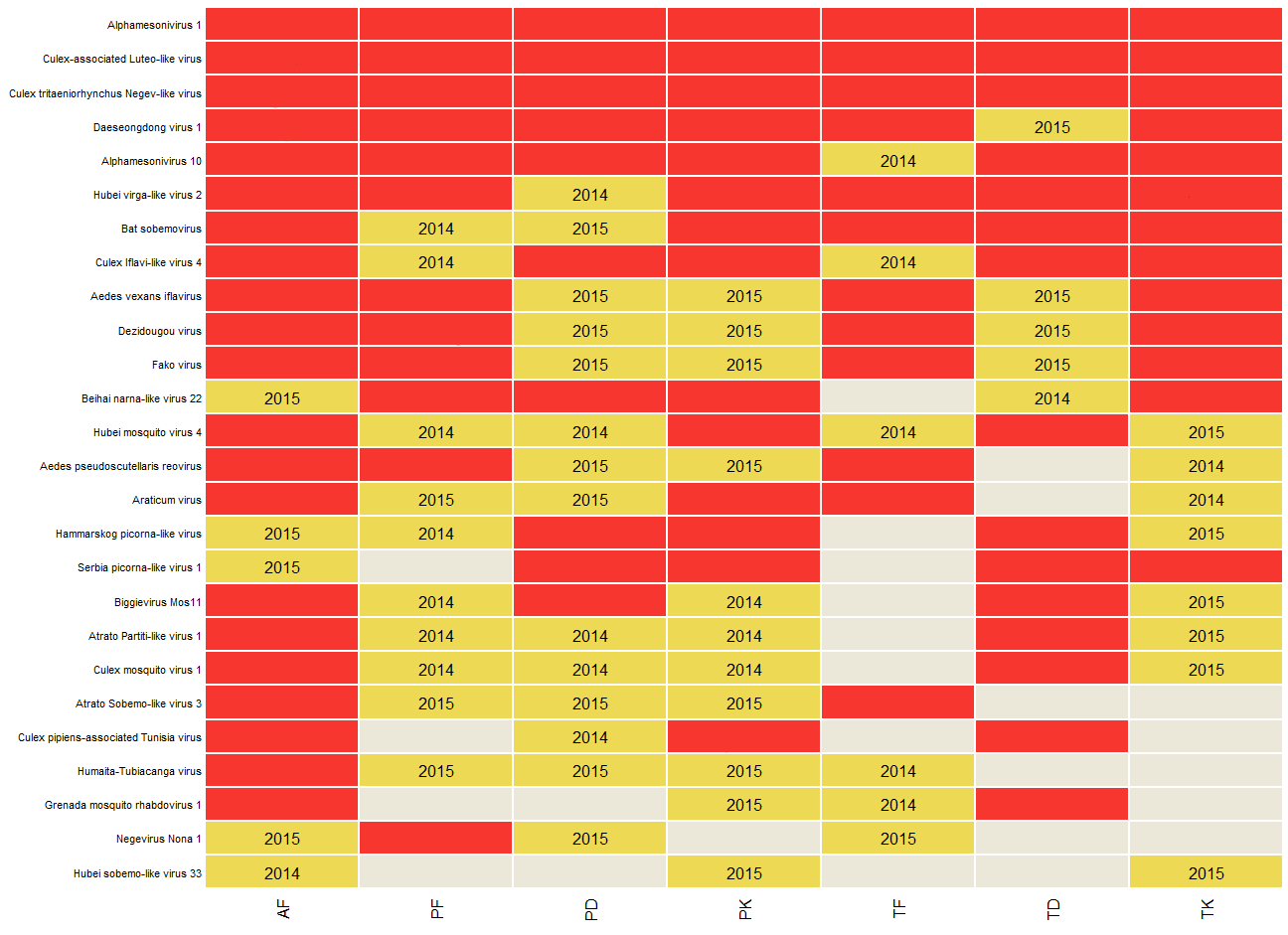

Supplement: S8 Fig — The VTUs are named after their best hit on the y axis. Each combination of mosquito species and site is presented on the x axis. Labels for the mosquito/site combinations are coded with the first letter standing for mosquito species (A for Aedes vexans, P for Culex poicilipes and T for Culex tritaeniorhynchus), and the second letter for site (F for the Ferlo Region (Ponds), D for the Diama village (River) and K for the Keur Momar Sarr village (Lake)). Tile color stands for number of years with detection in a mosquito/site combination (red: Two years, yellow: One year, gray: No detection). The year of detection is provided within the tile whenever the virus was detected only one year. (TIF) [file pone.0300915.s009.tif]
